# O’Reilley on the Placenta and Nervous System

**Published:** 1860-10

**Authors:** 


O'Reilley on the Placenta and Nervous System.

This recent little book consists of a series of papers published from time to time in the *American Medical Gazette*, entitled, "The Anatomy and Physiology of the Placenta." "The connection of the nervous centers of animal and Organic life." This last subject is illustrated by vivisections, and is illustrative of the influence of the maternal organization upon that of the foetus through the placenta.

There is also a paper containing "observations on syphilitic iritis."

These papers are ingenious and elaborate arguments in favor of the opinions of the author, as to the nature of the structure and functions of the placenta.

Although these opinions differ very decidedly in many respects from the doctrines of the present day, and come nearer a representation of those held by Hyppocrates than Coste, Cazeau and other modern accepted authors; Dr. O'Reilley has shown a zeal and tenacity in favor of them, which make us believe in his honesty of purpose.

